# Evidenzbasiert und praxisnah: die neue Leitlinie zur Thrombozytentransfusion im Fokus

**DOI:** 10.1007/s00101-025-01603-9

**Published:** 2025-11-03

**Authors:** Philipp Pütz, Mark Coburn, Florian Piekarski

**Affiliations:** Klinik für Anästhesiologie und Operative Intensivmedizin, Universitätsklinikum Bonn, Venusberg-Campus 1, 53127 Bonn, Deutschland

**Keywords:** Blutung, Thrombozytenkonzentrat, Restriktive Transfusionsstrategie, Thrombozytopenie, Transfusionsrichtlinien, Haemorrhage, Platelet concentrate, Restrictive transfusion strategy, Thrombocytopenia, Transfusion guidelines

## Abstract

Die Transfusion von Thrombozytenkonzentraten ist eine etablierte Therapie bei Thrombozytopenie oder Thrombozytenfunktionsstörungen. Die neue internationale Leitlinie der Association for the Advancement of Blood and Biotherapies in Zusammenarbeit mit der International Collaboration for Transfusion Medicine Guidelines basiert auf 21 randomisierten und 13 Beobachtungsstudien, in denen restriktive und liberale Transfusionsstrategien verglichen wurden. Die Evidenz zeigt, dass restriktive Strategien weder die 30-Tages-Mortalität noch das Risiko schwerer Blutungen (WHO-Grad 3–4) signifikant erhöhen, jedoch mit einer geringeren Rate transfusionsassoziierter Nebenwirkungen, einer verbesserten Ressourcenverfügbarkeit und niedrigeren Kosten einhergehen. Die Leitlinie unterstützt restriktive Transfusionsstrategien, betont jedoch die Notwendigkeit individueller klinischer Entscheidungen unter Berücksichtigung von Symptomen, Komorbiditäten und Patientenpräferenzen.

## Abstract (en)

The transfusion of platelet concentrates is an established treatment for thrombocytopenia or platelet dysfunction. The new international guideline from the Association for the Advancement of Blood and Biotherapies in collaboration with the International Collaboration for Transfusion Medicine Guidelines is based on 21 randomized and 13 observational studies comparing restrictive and liberal transfusion strategies. The evidence shows that restrictive strategies do not significantly increase 30-day mortality or the risk of severe bleeding (WHO grade 3–4), but are associated with a lower rate of transfusion-associated adverse events, improved resource availability, and lower costs. The guideline supports restrictive transfusion strategies, but emphasizes the need for individual clinical decisions taking into account symptoms, comorbidities, and patient preferences.

## Zusammenfassung der Leitlinie

Die Transfusion von Thrombozytenkonzentraten (TK) stellt eine etablierte therapeutische Maßnahme bei Patient*innen mit Thrombozytopenie oder Thrombozytenfunktionsstörungen dar [1]. Hinsichtlich des Nutzen-Risiko-Verhältnisses von TK-Transfusion und des Risikos für Blutungskomplikationen bestehen noch Unsicherheiten [2]. Trotz des bekannten Risikos transfusionsassoziierter Nebenwirkungen hat sich die Zahl der Thrombozytentransfusionen im Vergleich zu den Transfusionen von Erythrozytenkonzentraten (EK) in den letzten Jahren jedoch nicht signifikant reduziert [4–6].

Die aktuelle internationale Leitlinie, herausgegeben von der *Association for the Advancement of Blood and Biotherapies *(AABB) in Zusammenarbeit mit der *International Collaboration for Transfusion Medicine Guidelines* (ICTMG), basiert auf der Auswertung von 21 randomisierten kontrollierten Studien sowie 13 Beobachtungsstudien [3]. In diesen wurden restriktive und liberale Transfusionsstrategien systematisch miteinander verglichen.

Die Leitlinie enthält Empfehlungen zu prophylaktischen und prozedurbezogenen Thrombozytentransfusionen, u. a. bei hypoproliferativer Thrombozytopenie, invasiven Eingriffen (z. B. Lumbalpunktion, ZVK-Anlage, interventionsradiologischen Verfahren), neonataler Versorgung und spezifischen Krankheitsbildern wie z. B. Denguefieber.

Eine vollständige Übersicht der Empfehlungen, einschließlich Schwellenwerten und Evidenzsicherheit, findet sich in Tab. 1.

**Tab. 1:** Empfehlungen für die Thrombozytentransfusion

Klinische Situation	Thrombozytenschwelle		Empfehlung	Evidenzniveau	
**Starke Empfehlung**					
*1.1 Hypoproliferative Thrombozytopenie: Chemotherapie oder allogene SZT, keine Blutung*	< 10 ∙ 10^3^/μl		Transfundieren	Moderat	
*1.2 Frühgeborene ohne schwere Blutung*	< 25 ∙ 10^3^/μl		Transfundieren	Hoch	
*1.3 Vor Lumbalpunktion*	< 20 ∙ 10^3^/μl		Transfundieren	Moderat	
*1.4 Denguevirusinfektion-assoziierte Thrombozytopenie, ohne schwere Blutung*	–		Keine Transfusion	Moderat	
**Bedingte Empfehlungen**					
*2.1 Hypoproliferative Thrombozytopenie: autologe SZT oder aplastische Anämie, keine Blutung*	–		Keine Prophylaxe	Niedrig bis sehr niedrig	
*2.2 Verbrauchsbedingte Thrombozytopenie: aufgrund kritischer Erkrankung, ohne Blutung (Nichtdenguefieber)*	< 10 ∙ 10^3^/μl		Transfundieren	Sehr niedrig	
*2.3 ZVK-Anlage (komprimierbare Stelle)*	< 10 ∙ 10^3^/μl		Transfundieren	Moderat bis sehr niedrig	
*2.4 Interventionsradiologie*					
Niedrigrisikoverfahren	< 20 ∙ 10^3^/μl		Transfundieren	Sehr niedrig	
Hochrisikoverfahren	< 50 ∙ 10^3^/μl		Transfundieren	Sehr niedrig	
*2.5 Große nichtneuroaxiale Operation*	< 50 ∙ 10^3^/μl		Transfundieren	Sehr niedrig	
*2.6 Herzchirurgie ohne Thrombozytopenie/schwere Blutung*	–		Keine Transfusion	Sehr niedrig	
*2.7 ICB ohne chirurgischen Eingriff, auch unter TAH*	> 100 ∙ 10^3^/μl		Keine Transfusion	Sehr niedrig	

*SZT* Stammzelltransplantation, *ICB* intrakranielle Blutung, *TAH* Thrombozytenaggregationshemmer

Die enthaltene Metaanalyse zeigt mit mäßiger bis hoher Evidenz, dass restriktive Transfusionsstrategien wahrscheinlich nicht zu einem signifikanten Anstieg der 30-Tages-Mortalität führen (ARD: −0,4 %; 95 %-KI: −2,2 bis 1,7 %). Ebenso besteht kein relevanter Unterschied bei schweren Blutungen (WHO-Grade 3 und 4) (ARD: 0,3 %; 95 %-KI: −1,9 bis 3,0 %). Für klinisch relevante Blutungen (WHO-Grade 2–4) zeigt sich dagegen ein moderater Anstieg (ARD: 6,8 %; 95 %-KI: 0,9–12,8 %).

Restriktive Strategien führen darüber hinaus zu einer Reduktion transfusionsassoziierter Nebenwirkungen, einer besseren Verfügbarkeit von Thrombozytenkonzentraten und zu einer Senkung der Behandlungskosten [4, 14]. Eine Übersicht zu den transfusionsassoziierten Risiken ist der Leitlinie entnommen und in Tab. 2 dargestellt.

**Tab. 2:** Risiko von transfusionsbedingten unerwünschten Ereignissen

Art der Reaktion	Rate pro Transfusionsepisode	Rate pro TK-Transfusion	NNH
Allergisch [11]	NA	10–30/1000	33–1000 Einheiten
Anaphylaktisch [11]	NA	0,02–0,05/1000	20.000–50.000 Einheiten
Febril, nichthämolytisch [11]	NA	1–10/1000	100–1000 Einheiten
Septisch [7]	NA	< 0,1/1000	10.000 Einheiten
TACO	6,6/1000 [8] 8/1000 [9]	2,6/1000 [8]	385 Einheiten [8] 125 Episoden [9]
TRALI	0,8/1000 [9]	0,03/1000 [10]	33.333 Einheiten [9] 1250 Episoden [10]

*TACO* „transfusion-associated circulatory overload“, *TRALI* „transfusion-related acute lung injury“, *NA* nicht angegeben, *NNH* „number needed to harm“

Die zentralen Empfehlungen und praxisrelevanten Neuerungen der Leitlinie werden im vorliegenden Beitrag anhand eines klinischen Fallbeispiels dargestellt und diskutiert.

## Kommentar zur Leitlinie

Empfohlen wird ein restriktives Vorgehen bei Thrombozytentransfusionen. Ein Vorteil liberaler Transfusionsstrategien ließ sich in der aktuellen Evidenzlage nicht zuverlässig nachweisen. Die Leitlinie weist jedoch, wie auch frühere Empfehlungen [15], einige Einschränkungen auf: Patienten mit einer Thrombozytopenie bilden eine äußerst heterogene Population hinsichtlich des individuellen Blutungsrisikos. Diese Heterogenität wird möglicherweise nicht adäquat durch die Einschlusskriterien der berücksichtigten Studien oder die verfügbaren Ausgangsdaten abgebildet. Insbesondere für einige spezifische Subgruppen wie Patienten mit kardiochirurgischen Eingriffen oder nichtoperativen intrakraniellen Blutungen basiert die Evidenz auf kleinen Studien oder Beobachtungsdaten mit niedriger bis sehr niedriger Evidenzsicherheit. Die Leitlinie enthält ferner keine generelle Empfehlung zur Gabe von Thrombozytentransfusionen bei Patienten unter Thrombozytenaggregationshemmung. Sie bezieht sich hierzu ausschließlich auf den Kontext der nichtoperativen intrakraniellen Blutung mit Thrombozytenzahlen > 100 ∙ 10^3^/µl und empfiehlt in dieser Situation keine prophylaktische Transfusion.

## Fallbeispiel: Frau M., 64 Jahre, AML unter Chemotherapie

### Anamnese und Befund

Frau M., 64 Jahre, wird aufgrund einer akuten myeloischen Leukämie (AML) im Rahmen der Induktionstherapie stationär behandelt. Die Patientin ist hämodynamisch stabil, ohne Anzeichen für eine aktive Blutung, und weist eine Thrombozytenzahl von 42 ∙ 10^3^/µl auf.

Es ist eine Lumbalpunktion zur Diagnostik eines fieberhaften Infekts geplant. Außerdem soll ein zentraler Venenkatheter (ZVK) über die V. jugularis interna neu gelegt werden. Im Verlauf ist eine interventionsradiologische Leberbiopsie vorgesehen.

### Klinische Fragestellungen und Empfehlungen gemäß Leitlinie

#### Prophylaktische Thrombozytentransfusion bei hypoproliferativer Thrombozytopenie (HPT).

Empfehlung 1.1 (stark): Bei nichtblutenden Patienten mit HPT (z. B. AML unter Chemotherapie) ist eine Transfusion bei < 10 ∙ 10^3^/μl empfohlen.

Anwendung: Frau M. liegt mit 42 ∙ 10^3^/μl oberhalb dieser Schwelle, eine prophylaktische Transfusion ist daher nicht erforderlich.

#### Lumbalpunktion bei schwerer Thrombozytopenie.

Empfehlung 1.3 (stark): Bei geplanter Lumbalpunktion ist eine Transfusion bei < 20 ∙ 10^3^/μl indiziert.

Anwendung: Da Frau M. mit 42 ∙ 10^3^/μl oberhalb dieser Grenze liegt, ist keine Transfusion notwendig.

#### ZVK-Anlage an komprimierbarer Stelle.

Empfehlung 2.3 (bedingt): Bei Anlage eines ZVK an einer komprimierbaren Stelle (z. B. V. jugularis) ist eine Transfusion bei < 10 ∙ 10^3^/μl empfehlenswert.

Anwendung: Frau M. liegt deutlich oberhalb dieser Schwelle; eine Transfusion ist nicht indiziert.

#### Interventionsradiologie – Leberbiopsie.

Empfehlung 2.4 (bedingt): Eine Leberbiopsie gilt als hochrisikoreiches Verfahren; der Schwellenwert liegt laut Leitlinie bei < 50 ∙ 10^3^/μl.

Anwendung: Mit 42 ∙ 10^3^/μl liegt Frau M. unterhalb des empfohlenen Schwellenwerts, sodass in diesem Fall eine Transfusion erforderlich ist.

Gemäß Leitlinie ist weder eine prophylaktische Thrombozytentransfusion noch eine im Rahmen einer Lumbalpunktion oder ZVK-Anlage erforderlich. Eine Indikation besteht ausschließlich für die geplante Leberbiopsie aufgrund des erhöhten Blutungsrisikos. Die weiteren Empfehlungen der Leitlinie finden sich in Tab. 1 zusammengefasst.

## Fazit für die Praxis

- Die Leitlinie befürwortet restriktive Strategien für die Thrombozytentransfusion.
- Die Studienlage zeigte keine konsistenten Belege für den Vorteil von liberalen Transfusionsstrategien in Bezug auf die klinischen Ergebnisse.
- Neben den evidenzbasierten Empfehlungen betont das Expertengremium die Bedeutung individueller klinischer Beurteilung unter Berücksichtigung von Symptomen, Komorbiditäten und Patientenpräferenzen.
